# Dr. Robert Battey

**Published:** 1895-12

**Authors:** 


Dr. Robert Battey, of Rome, Ga., died at his residence, November 8, 1895, aged 67 years. He received his preliminary education at Augusta, Ga., and Phillips's academy, Andover, Mass., and took his doctorate degree at Jefferson Medical College, March 7, 1857. From November, 1872, to October, 1875, he was professor of obstetrics at the Atlanta Medical College and was editor of the *Atlanta Medical and Surgical Journal.* He was gifted as a physician and an ingenious surgeon, having devised many original methods for the treatment of disease. His greatest claim to renown, however, consists in an operation that he devised to effect the change of life in women by removal of the ovaries, its object being to modify or cure certain diseases otherwise beyond remedy. Besides membership in his local societies, city, county and state, he was a Fellow of the American Gynecological Society and a member of the American Medical Association. He served in the medical department of the Confederate army from the beginning to the end of the war. Dr. Battey was a courteous gentleman, an able physician and died respected by his fellow-citizens and his professional colleagues.

